# Dr. Middleton Michel

**Published:** 1894-07

**Authors:** 


DR. MIDDLETON MICHEL, of Charleston, S. C., died June 4th, at the age of seventy-two years. Dr. Michel was a native of Charleston and graduated from the Medical College of South Carolina. A part of his medical education was also received in Paris. Dr. Michel was for many years Professor of Physiology in the Medical College of South Carolina, and one of the most successful, accomplished and popular teachers in the South. He once conducted a "Summer Institute," consisting mainly of lectures on anatomy and physiology, which were largely attended. During the late war he was a medical officer in the Confederate army. He had made many contributions to medical literature, chiefly in the fields of physiology and obstetrics.

